# Early signs of lameness in dairy cows revealed by milking and behavioral data using generalized additive mixed models

**DOI:** 10.1093/jas/skag289

**Published:** 2026-09-08

**Authors:** Sara Mondini, Chris Major Ncho, Giulia Gislon, Anna Sandrucci, Stefano Cossa, Maddalena Zucali

**Affiliations:** Department of Agricultural and Environmental Sciences, University of Milan, Milan 20133, Italy; Animal Nutrition, Institute of Agricultural Sciences, Department of Environmental Systems Science, ETH Zürich, Zürich 8092, Switzerland; Department of Agricultural and Environmental Sciences, University of Milan, Milan 20133, Italy; Department of Agricultural and Environmental Sciences, University of Milan, Milan 20133, Italy; Società Agricola Cossa, Buccinasco 20090, Italy; Department of Agricultural and Environmental Sciences, University of Milan, Milan 20133, Italy

**Keywords:** milk ejection, milk flow, milking duration, steps, lying time

## Abstract

Early detection of lameness is difficult in dairy farms as clinical signs may be subtle. This study aims to identify key changes in milking and behavioral data before and after lameness identification, to assess their potential as early-warning indicators. Data were collected on a dairy farm in Northern Italy, with 600 Holstein cows milked twice daily in a rotary parlor, equipped with sensors and pedometers. A total of 67 cows (days in milk: 177 ± 104; parity: 2.62 ± 1.57) with at least one farmer-detected lameness event between 2022 and 2023 were included. Daily data on milk yield, duration, flows, lying time, bouts, and steps were analyzed in a 60-d window around the detection day (Δ –30 to +30), using generalized additive mixed models (GAMMs) accounting for parity and stage of lactation. Daily milk yield began to decline 12–16 d before detection, with losses of up to 16% in multiparous cows. Before lameness detection, milking duration shortened, and average milk flow slowed in multiparous cows, particularly between 60 and 120 s after cluster attachment. Lying time increased by 8–11% in the days before detection, while steps differed by parity: primiparous cows reduced their activity, whereas multiparous cows showed increased steps. After hoof trimming, most of the traits returned toward pre-lameness levels. Although the magnitude of changes was moderate, timing and consistency indicate that deviations in milking performance and behavior can be detected before lameness becomes visible. These findings provide a basis for developing predictive algorithms to identify cows at risk earlier, using standard farm infrastructure, without additional sensors.

## Teaser

Generalized additive mixed models can be used to describe milking and behavior changes around a lameness event. The trends highlighted that most parameters decline (e.g. daily milk and milking duration) or increase (e.g. lying time) up to 20 d before lameness detection. This suggests future possibilities to train and test algorithms for the detection of lameness in an early way using only on-farm data.

## Graphical abstract

**Graphical Abstract:**
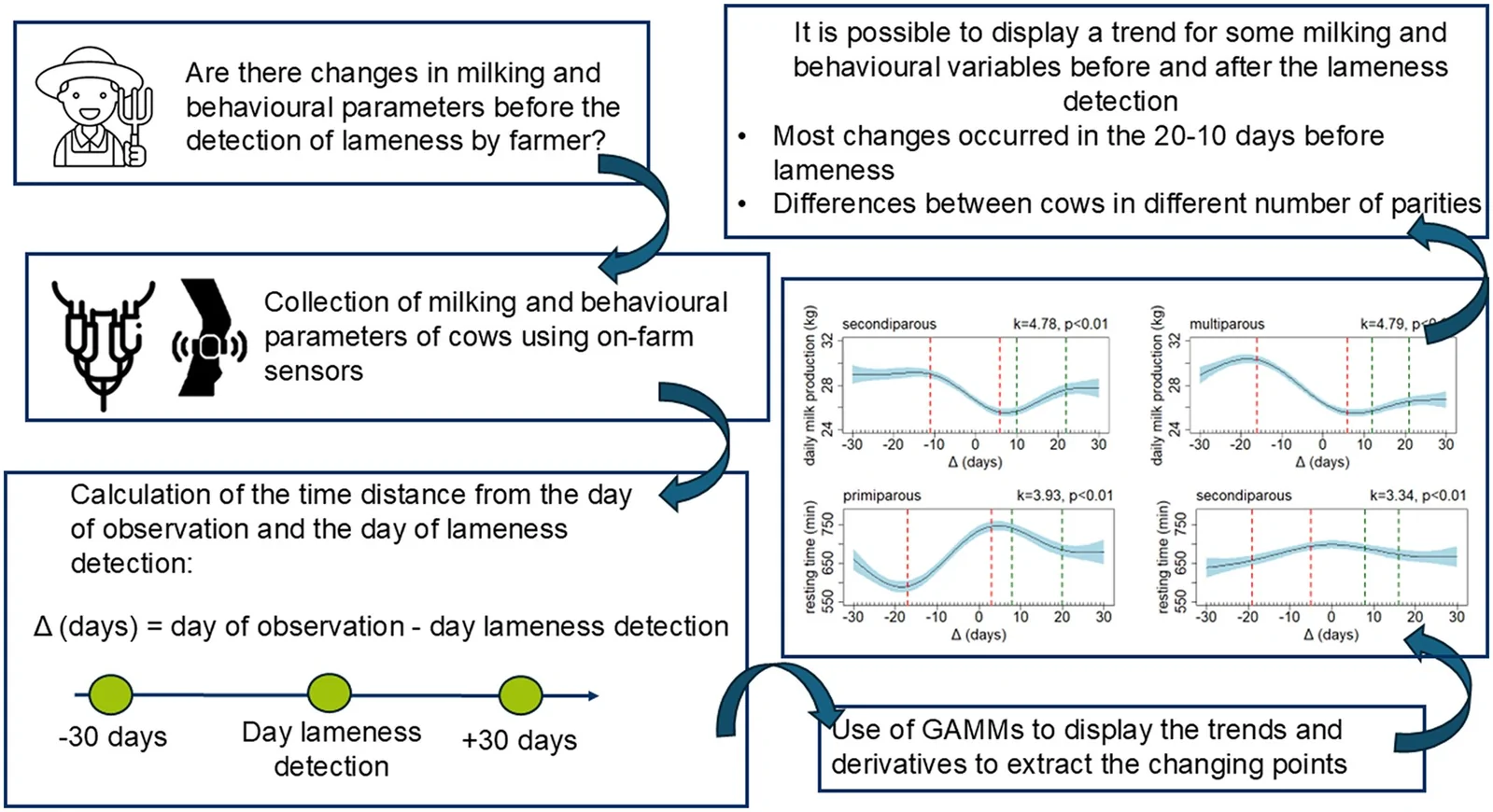

## Introduction

Lameness is one of the most significant health and welfare issues in dairy farms, alongside mastitis and reproductive disorders. Recent studies report a prevalence of around 20–30% in Europe and North America, with severe cases affecting about 7% of cows (Thomsen et al. 2023; Roche et al. 2024). Lameness is a multifactorial disease, with environmental, nutritional, genetic and physiological or pathological factors often acting together (Singh et al. 2005; Refaai et al. 2013). Its consequences include decreased milk production, impaired fertility, higher culling rates (Green et al. 2002; Melendez et al. 2003; Cramer et al. 2009) and associated economic losses (Huxley 2013; Puerto et al. 2021), These effects are also associated with an increase in the environmental impact of milk production (Bianchi et al. 2024).

Lameness is defined as “a deviation in gait to reduce pain” (Scott 1989) and is associated with altered cow behavior. Lame cows spend more time lying down and less time walking or standing (Weigele et al. 2018) with a consequent reduction in feeding and ruminating (Miguel-Pacheco et al. 2014; Norring et al. 2014; Beer et al. 2016). These changes could be associated with the reduced milk production during lameness episodes. An earlier study reported milk losses of about 360 kg per lactation (Green et al. 2002), whereas a more recent study estimated 800–1,300 kg depending on lactation stage (Puerto et al. 2021).

Moreover, not only does milk production seem to be affected by lameness, but also milk ejection. Wieland et al. (2022) identified the presence of lameness as one of the risk factors for delayed milk ejection during milking, and these results also agree with the study of Juozaitienė et al. (2021), which observed a lower emission in the first minute of milking and a delayed plateau phase in lame cows. One possible explanation is that both studies observed a higher concentration of blood cortisol in lame cows, due to stress and pain, compared to healthy animals. Bruckmaier (2005) reported that cortisol is an antagonist of oxytocin, and elevated concentrations of cortisol may reduce or slow the release of oxytocin, disrupting milk ejection.

Despite its impact, early detection of lameness remains a major challenge under farm conditions, as clinical signs may be subtle and routine locomotion scoring is often inconsistent or infrequent. Although several Precision Livestock Farming (PLF) technologies have been developed to detect lameness at a research level (Alsaaod et al. 2019; Taneja et al. 2020), only a few systems are currently available on the market for early-stage detection (e.g. CattleEye Ltd.). These commercial solutions require additional sensors, high implementation costs, and their effectiveness in practical farm conditions is still limited and needs further validation. This raises the question of whether early signs of lameness could be detected by using data from PLF tools that are already widely adopted in dairy farms, such as milking system sensors and pedometers. Identifying behavioral and milking-related changes from existing infrastructure could represent a more accessible and scalable strategy for proactive lameness management.

Previous studies described the difference between lame and healthy cows in terms of milking traits or behavioral patterns (Miguel-Pacheco et al. 2014; Norring et al. 2014; Beer et al. 2016), but only a few studies (King et al. 2017; Zhou et al. 2022) have described how the changes develop in the days preceding lameness detection, considering this disease together with other health disorders or analyzing only milking or behavioral parameters separately.

On the other hand, physiological and behavioral responses of animals to health and welfare issues have been investigated using various modeling techniques. Recently, the application of generalized additive models (GAMs) and their extension to generalized additive mixed models (GAMMs) has garnered significant attention from researchers in the dairy industry (Stocco et al. 2025). These models facilitate the depiction of the relationship between explanatory variables and responses through smooth curves, which are penalized to prevent overfitting (Pedersen et al. 2019). This characteristic of GAMMs makes them particularly effective in capturing the complex relationships and nonlinear patterns inherent in milk production (Biffani et al. 2025), disease occurrence (Ankinakatte et al. 2013), and the behavior (Benni et al. 2020) of dairy animals. Indeed, a previous study successfully employed GAMMs to describe the behavioral adaptation of dairy cows during hot summers (Ferrari et al. 2025). Similarly, the use of GAMMs in examining the relationship between somatic cell properties and milk production revealed distinct trends between primiparous and multiparous cows (Huang et al. 2023). Therefore, in the current context, the application of GAMMs is not only beneficial for accurately modeling milking traits and behavioral patterns but also may provide early insights into the occurrence of health issues, notably lameness.

This study aims to identify key changes in milk yield, milk flows, milking duration, lying time, and activity patterns in the 30 d before and after farmer-detected lameness. These changes are modeled within GAMMs, under the hypothesis that deviations in milking and behavioral parameters precede clinical signs by several days, thus holding potential as early-warning indicators. To the best of our knowledge, no previous studies have tracked the dynamic changes in both milking traits and behavioral patterns in the weeks preceding clinical detection of lameness, and by using exclusively data from routine farm monitoring systems.

## Material and methods

### Ethics statement

The study was carried out in full compliance with animal welfare standards. It received a favorable opinion (OPBA_120_2022, June 30, 2023) from the Animal Welfare Body (OPBA) of the University of Milan. The data collection was limited to farm records (e.g. parity, lactation stage, milking data, and behavioral data), conducted without handling or physical interaction with animals.

### Farm description

Data were collected in a dairy cattle farm in Northern Italy (Lombardy Region). The farm consisted of about 600 adult Holstein cows, including 500 lactating and 100 dry cows. The average milk production in the study period was 31.4 kg/day/cow, with 3.81% of fat and 3.41% of protein in milk. The average parity number during the observation year was 2.4 ± 1.3, and primiparous cows represented 38% of the lactating herd. Lactating cows were housed in straw-bedded cubicles; dry cows were kept in a straw-bedded deep-litter pen with access to an outdoor paddock. During summer, fans were used for cooling. Cows were milked twice a day in a rotary milking parlor with 36 bails (Tecnozoo impianti s.r.l., Zelo Buon Persico, Lodi, Italy) equipped with the AfiMilk System (Afimilk Agricultural Cooperative Ltd., Kibbutz Afikim, Israel). The diet remained approximately the same throughout the year, using a total mixed ration (TMR): dry cows and primiparous lactating cows were fed once daily, and multiparous cows twice daily. The TMR for lactating cows consisted mainly of corn and grass silages, corn meal, protein and mineral concentrates, soybean extraction meal, and hay.

The diet for lactating cows (24 kg of dry matter/cow/day) was composed of 35% NDF and 15% crude protein, with 51% of forages on dry matter. Dry cows received a TMR of hay, sorghum silage, energy-rich by-products, and corn meal, with a final diet (13 kg of dry matter/cow/day) with 61% of NDF and 13.7% of crude protein and 79% of forages. The prevalence of lameness was around 15% and of mastitis 27%, calculated as a percentage of cows treated during the year (reported by the farmer) and the number of cows in the herd. A single farm was selected to reduce variability of factors (e.g. diet composition, management practices, and housing) that can cause lameness.

### Data collection

#### Lameness data

Data about lameness were recorded by the farmer from December 2022 to December 2023. The observation of the cows was performed every day during both daily milkings, while walking (entering the milking parlor) and standing (during cluster attachment), by farm operators trained during hoof-trimming courses. Cows that were visibly lame, with impairment in gait and inability to equally distribute pressure on all four legs, were separated immediately after milking and treated with a hoof trimmer intervention, and in the most severe cases, with antimicrobials. The main lamenesses detected and treated were digital dermatitis (85%) and sole ulcers (15%), and the recurrence cases were 26%. The farm operators recorded the date of lameness detection and treatment. The same cows could have been recorded lame several times during the same year. A limitation of the present study is that detection of lameness can be subjective, and even though farm operators were trained in the same way, personal bias may impact the internal and external validity.

#### Milking and behavioral data

Milking and behavioral data were collected for one entire year, from January 1, 2023 to December 31, 2023. AfiMilk sensors (Afimilk Agricultural Cooperative Ltd., Kibbutz Afikim, Israel) on the milking parlor collected data about milk production (kg), milk production in the first 2 min of milking (as kg and as % of milk production), milking duration (min), average milk flow (kg/min) and milk flows (kg/min) in four intervals (0–15 s, 15–30 s, 30–60 s and 60–120 s). AfiMilk pedometers (Afimilk Agricultural Cooperative Ltd., Kibbutz Afikim, Israel), validated by Borchers et al. (2016) and Marques et al. (2024), were used to collect data about lying time (min), number of lying bouts (n), average lying bout duration (min), and hourly steps (n); besides the percentage of lying time (%) was calculated from raw data. Each milking data point was referred to the milking of the day for each cow. Behavioral data were referred to the time distance between milkings (around 12 h). Each cow had two records per day, composed of milking and behavioral data.

### Data handling and quality control

The starting dataset consisted of 374,130 milking and behavioral records from all 600 adult cows of the herd. At the first step, observations with missing milking or behavioral variables were deleted. At the second step, the dataset was cleaned of outliers: milk production per milking <3 kg or >40 kg, average milking duration <3 min, milking flows in the four intervals >10 kg/min and milking flow at 60–120 s <0.1 kg/min, bout number = 0, average lying time <3 min and average lying time per bout >500 min. The cleaned dataset was composed of 293,511 observations on the same cows. This dataset was merged with the dataset of lameness detection in 2022–2023, and a new variable was calculated:


Δ (days)=date of observation -date of lameness detection

If the day of observation was before the day of lameness detection, Δ values were negative, and positive if the observation was recorded after the date of lameness detection. If no lameness occurred in the trial period, Δ was coded as NA.

To describe changes in milking traits and behavioral patterns around lameness, a subset of only lame cows (at least one lameness in the trial period) was extracted (*n* = 92 cows). Considering the literature, the signals of lameness, as changes in milk production and behavior, start 2 wk before the clinical evidence (Rajala-Schultz et al. 1999), and the effects can continue for more than 3 wk (Warnick et al. 2001; Chapinal et al. 2010). So, only observations in the interval −30 ≤ Δ ≤ +30 d were considered, and only cows with at least 30 complete observations (composed of milking and behavioral parameters) in this window were considered. Cows with 3 or more lameness/mastitis events in the trial period were excluded to avoid including animals that were chronically problematic and had likely not fully recovered from the previous lameness event. Moreover, cows with one clinical/subclinical mastitis event, defined through visual observation, Somatic Cell Count, microbial cultures or veterinary diagnosis, within 60 d before or in the 30 d after lameness detection were deleted, because the variability of milking traits and behavioral indicators could be associated with mastitis and not lameness. Similarly, all the observations of cows with two or more hoof trimmer interventions within 60 d (indicating unresolved lameness) were deleted. Observations with more than 400 d in milk were also deleted.

At the end, the milking and behavioral data of each cow were summarized in a daily observation (mean or sum of the two milkings). The new variables are described in Table 1. At last, if only one milking data point per day was recorded, the observation was deleted. The final daily dataset included 3,459 observations from 67 cows (18 primiparous, 22 secondiparous, and 27 multiparous). Days in milk (DIM) were categorized into early lactation (<100 DIM), mid lactation (100–250 DIM), and late lactation (>250 DIM). The distribution was as follows: among primiparous cows, 8 were in early lactation, 6 in mid lactation, and 4 in late lactation; among second-parity cows, 7 were in early lactation, 10 in mid lactation, and 5 in late lactation; among multiparous cows, 12 were in early lactation, 9 in mid lactation, and 6 in late lactation.

**Table 1:** Daily milking traits and behavioral indicators derived from sensor data and used in statistical models, calculated as the sum or the average of the two records collected every day for each cow.

Daily variables	Unit	Calculation
**Individual daily milk production**	kg	Sum of two daily milkings
**Milking duration**	min	Mean duration of two daily milkings
**Milk flow**	kg/min	Mean of the two average milk flows of the day by cow
**Milk in the first 2 min**	kg	Average quantity of milk produced in the first 2 min
**Milk in the first 2 min**	%	Average percentage of milk produced in the first 2 min
**Milk flow between (0–15 s, 15–30 s, 30–60 s, 60–120 s)**	kg/min	Average milk flow in fixed intervals during milking
**Lying time**	min	Sum of two records of daily lying time
**Lying time**	%	Sum of two records of daily lying time divided by the minutes of the day
**Daily steps**	n	Sum of two records of the daily number of steps
**Bout number**	n	Sum of two records of daily bout numbers
**Lying time per bout**	min	Sum of two records of daily lying time, divided by the sum of two records of daily bout number

Lameness occurred in early lactation (starting at DIM <100 days) for 27 cows, in mid lactation (100 < DIM <250) for 25 cows, and in late lactation (>250 DIM) for 15 cows.

### Statistical analysis

The dataset was analyzed using R software (version 2024.09.0). GAMMs, employing the *mgcv* package (Wood 2011), were used to describe the changes in milking traits and behavioral indicators around lameness events.

The following model was applied to each dependent variable (individual daily milk production (kg), milking duration (min), average milk flow (kg/min), milk in first 2 min (kg and %4), milk flow 0–15 s (kg/min), milk flow 15–30 s (kg/min), milk flow 30–60 s (kg/min), milk flow 60–120 s (kg/min), lying time (min and %), daily steps (n), bout number (n), and lying time per bout (min)):


$$y_{\mathrm{ijkl}}=\alpha +\mathrm{parit}y_{i}+\mathrm{seaso}n_{j}+f_{i}(\lambda_{\mathrm{ijkl}})+g_{i}(\mathrm{DI}M_{\mathrm{ijkl}})+u_{k}+\varepsilon_{\mathrm{ijkl}}$$

where *y*_*ijkl*_ is the observed value of the dependent variable for parity class *i*, season *j*, cow *k*, at observation *l*. *α* is the overall intercept. parity*_i_* is the parametric fixed effect of parity class (*i* = primiparous, secondiparous, multiparous). season_*j*_ is the parametric fixed effect of season (*j* = winter, spring, summer, autumn). *f_i_*(*λ*) is a parity-specific penalized thin-plate regression spline of days relative to lameness onset (λ = 0 at detection and treatment), estimated as a separate smooth for each level of parity *i* (*i* = primiparous, secondiparous, multiparous) via the by argument in *mgcv*. *g*_*i*_(DIM) is a parity-specific penalized thin-plate regression spline of days in milk, defined analogously to *f*_*i*_(λ). *u*_*k*_ ∼ N(0, σ^2^_u_) is the random intercept of cow *k*, accounting for repeated measures within animal. *ε*_*ijkl*_ ∼ N(0, σ^2^) is the residual error.

Considering that milking flows and milking duration could be defined as milk production-associated variables, the addition of individual daily milk production as a smooth term was tested. The deviance explained by these models was less than 3% of that of the model proposed in this section (Table S1). Hence, these models were not considered, as the improvement in deviance explained was not enough to justify an increase in model complexity.

Smoothing parameters were estimated by restricted maximum likelihood (REML). The parity-specific smooths were implemented as factor-smooth interactions using the by argument within *mgcv*, which fits a separate deviation smooth for each level of the parity factor. The random intercept *u_k_* was fitted as a random-effect smoother (bs = “re”), equivalent to a standard mixed model random intercept. The basis dimension *k* for each smooth term was verified using the diagnostic procedure of Wood (2017) to ensure adequate flexibility without overfitting.

Output of GAMM models was extracted using gam.summary, and residual diagnostic plots for each parameter were performed using gam.check to analyze the right application of the model (Figure S1). The residual diagnostic plots highlighted that for some parameters (e.g. milk production, milk production in the first 2 min of milking, lying time) the distribution of data was comparable to a Gaussian distribution. For other parameters (e.g. milk flows in the different slot intervals, daily steps, and bout numbers), a different family distribution (e.g. Tweedie or zero-inflated Poisson) of data was tested during model running (Figure S2), but the residual diagnostic plots also in this case were not optimized and comparable to the first ones using the Gaussian family. Moreover, the AIC values were lower with models using Gaussian families compared to other tested models for behavioral parameters, and the increase in variance explained did not justify the adoption of alternative distributions (Table S2). So, the Gaussian distribution of data was only an assumption for some parameters but was preferred to simplify result interpretations.

The *gratia* package (Simpson 2024) was used to identify the derivatives of the smooth term Δ at the 60 points in the mean and the 95% confidence interval. The function was defined as flat when the simultaneous confidence interval of the derivatives included 0. The changing points were defined as the first and last points of the smooth term, where all the confidence intervals of the derivatives were positive (ascending function) or all negative (descending function). Only the changing point nearest to Δ = 0 d was used in plots. The plot function of *mgcv* package was used to define the mean value of dependent variables at the changing points.

## Results

The descriptive statistics of the dataset are reported in Table 2.

**Table 2:** Descriptive statistics of daily milking and behavioral parameters.

	Mean	SD	Min	p5	Median	p95	Max
**General information**							
** Number of parities**	2.62	1.57	1	1	2	6	8
** Days in milk**	177	104	2	19	188	336	400
**Milking traits**							
** Individual daily milk production, kg**	30.0	6.36	7.10	19.2	30.0	41.1	50.6
** Milking duration, min**	7.56	3.36	3.30	4.15	6.40	15.3	19.7
** Average milk flow, kg/min**	2.31	0.89	0.55	1.00	2.20	3.75	5.80
** Milk in first 2 min, kg**	4.52	2.08	0.75	1.65	4.25	8.55	14.50
** Milk in first 2 min, %**	30.2	12.1	5.00	13.5	28.5	53.1	92.5
** Milk flow 0–15 s, kg/min**	0.26	0.43	0.00	0.00	0.00	0.93	4.61
** Milk flow 15–30 s, kg/min**	2.28	1.27	0.00	0.43	2.20	4.61	8.04
** Milk flow 30–60 s, kg/min**	1.85	1.18	0.00	0.43	1.67	3.93	8.03
** Milk flow 60–120 s, kg/min**	2.96	1.37	0.22	1.05	2.79	5.61	8.79
**Behavioral indicators**							
** Lying time, min**	700	162	84	432	702	960	1179
** Lying time, %**	49	11	6	30	49	67	82
** Daily steps, *n***	2,792	972	1,107	1,652	2,609	4,731	9,846
** Bout number, *n***	13.2	4.31	2.00	7.00	13.0	21.0	38.0
** Lying time per bout, min**	58	20	12	31	55	92	166

Output of GAMM models was summarized in three tables (Tables 3 and 4; Table S3), reporting for parametric coefficients the estimates, standard errors, *t*-values, and *P*-values for parity and season fixed effects, and for the smooth terms the effective degrees of freedom (edf), F-statistics, and *P*-values. In the tables for each model, the adjusted *R*^2^ and the deviance explained were reported. In Figures 1–4, the results of GAMM models for the smooth term of Δ are reported. The reported numerical values of the dependent variables were calculated at the changing points, and the decrease or increase referred to the period between two changing points. Details of Δ and derivatives of changing points were reported in Table S4.

**Figure 1:**
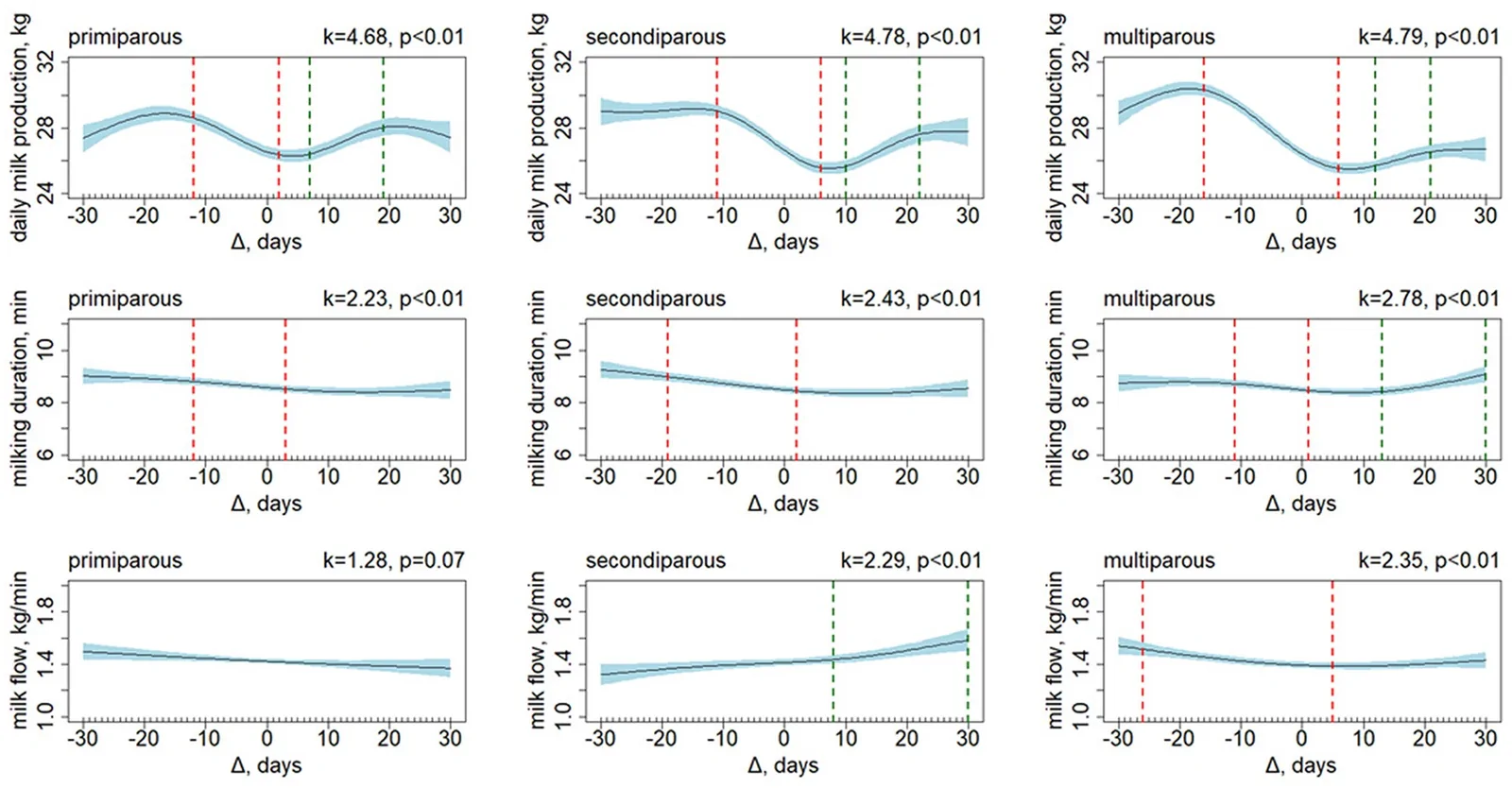
Effect of Δ on milk production, milking duration, and milk flow. Effect of Δ (time distance between the observation day and the day of lameness detection) on individual daily milk production, average milking duration, and average milk flow as fitted in a generalized mixed model for the three parity classes (primiparous, secondiparous, and multiparous). P identified the *P*-value of the smooth term. The blue shadows showed the 95% confidence interval for each point of the smooth. The red and green lines identified the changing point of the smooth, where the derivatives of the function were statistically different from 0 in the 95% confidence interval.

**Figure 2:**
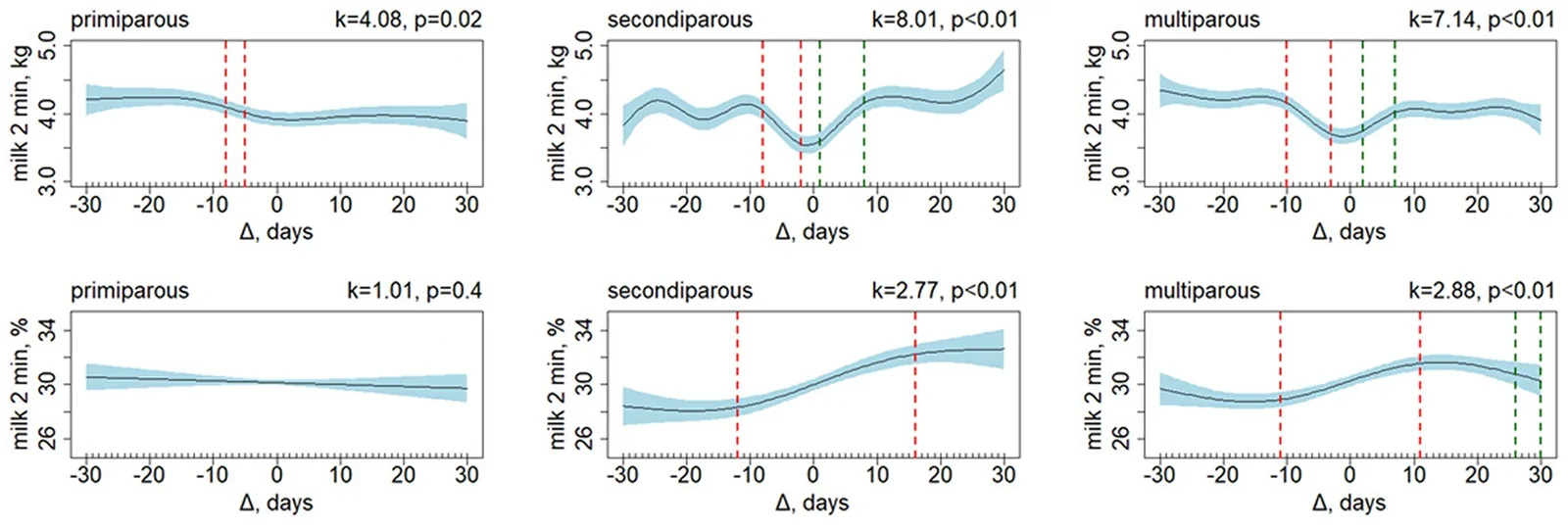
Effect of Δ on milk yield emitted in the first 2 min of milking. Effect of Δ (time distance between the observation day and the day of lameness detection) on milk yield emitted in the first 2 min of milking in kg and in % of the total milk production as fitted in a generalized mixed model for the three parity classes (primiparous, secondiparous, and multiparous). P identified the *P*-value of the smooth term. The blue shadows showed the 95% confidence interval for each point of the smooth. The red and green lines identified the changing point of the smooth, where the derivatives of the function were statistically different from 0 in the 95% confidence interval.

**Figure 3:**
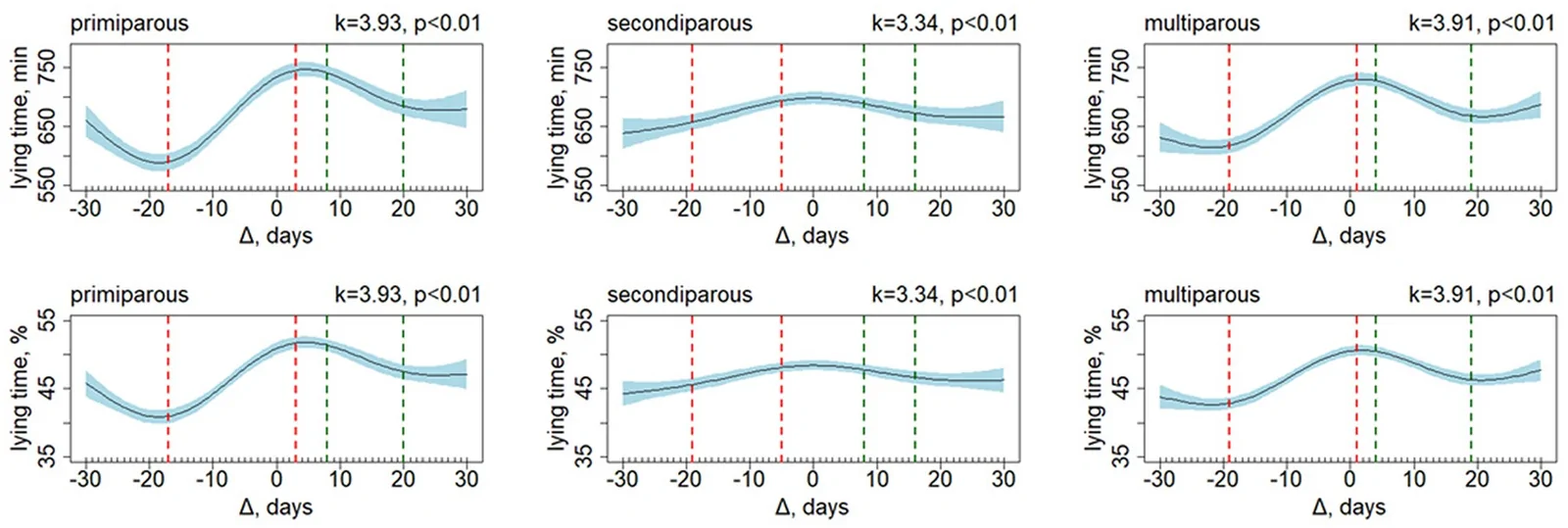
Effect of Δ on daily lying time. Effect of Δ (time distance between the observation day and the day of lameness detection) on daily lying time in minutes and in % of the day duration as fitted in a generalized mixed model for the three parity classes (primiparous, secondiparous, and multiparous). P identified the *P*-value of the smooth term. The blue shadows showed the 95% confidence interval for each point of the smooth. The red and green lines identified the changing point of the smooth, where the derivatives of the function were statistically different from 0 in the 95% confidence interval.

**Figure 4:**
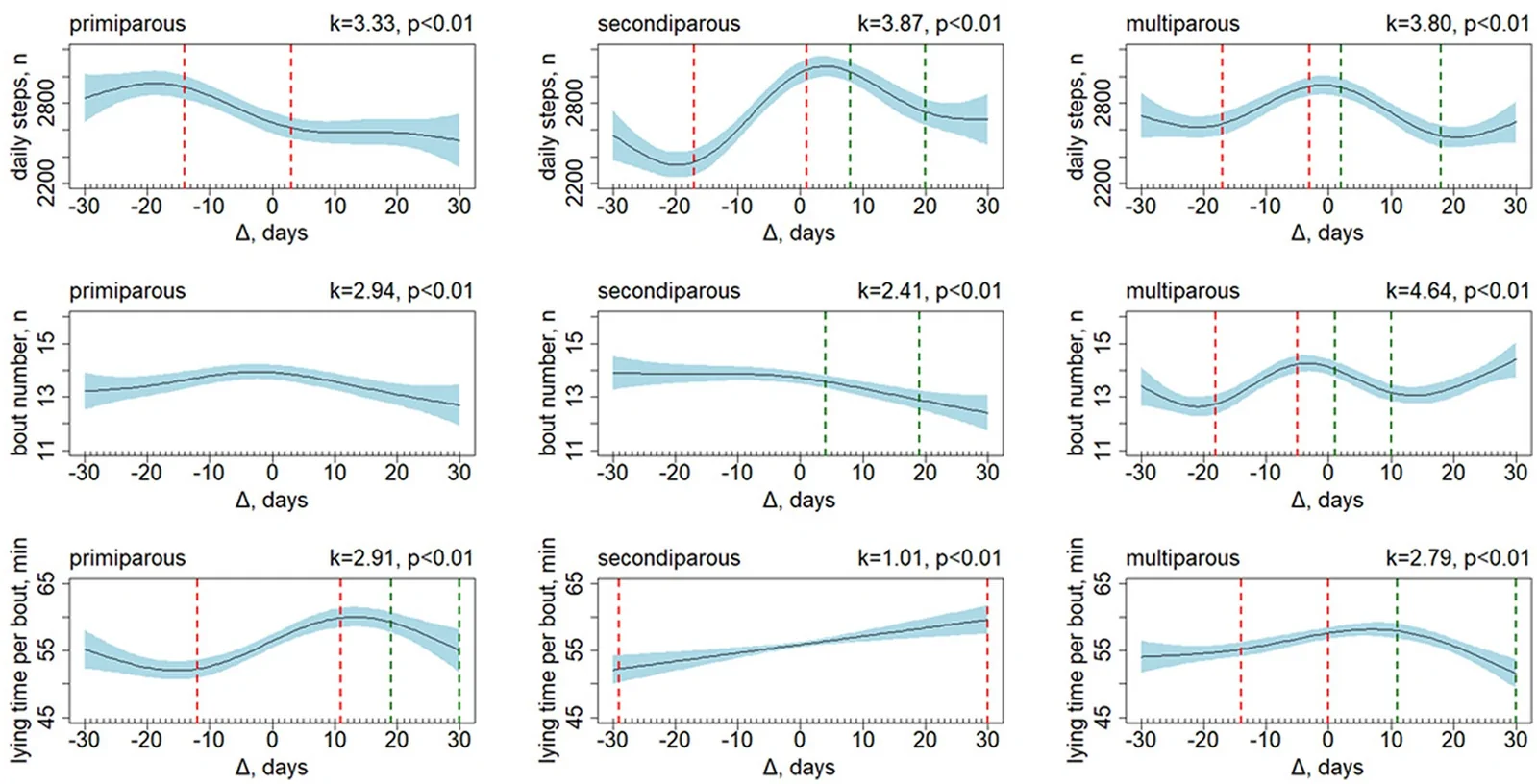
Effect of Δ on number of steps, number of bouts, and lying time per bout. Effect of Δ (time distance between the observation day and the day of lameness detection) on daily number of steps, daily number of bouts and average lying time per bout as fitted in a generalized mixed model for the three parity classes (primiparous, secondiparous, and multiparous). P identified the *P*-value of the smooth term. The blue shadows showed the 95% confidence interval for each point of the smooth. The red and green lines identified the changing point of the smooth, where the derivatives of the function were statistically different from 0 in the 95% confidence interval.

**Table 3:** Results of GAMM models on milking parameters for parametric coefficients (estimates, standard errors (SE), *t*-values, and *P*-values for parity and season fixed effects), and for smooth terms (effective degrees of freedom (edf), F-statistics, and *P*-values). For each model, the adjusted *R*^2^ and the deviance explained were reported. In the section on parametric coefficients, the estimates referred to the difference from a reference: multiparous for parity effect and spring for season effect.

		Individual daily milk production	Milking duration	Average milk flow	Milk in first 2 min as kg	Milk in first 2 min as %
**Parametric coefficients**						
** Intercept**	Estimate	27.66	8.64	1.43	4.05	30.15
	SE	0.90	0.52	0.15	0.31	2.05
	*t*-value	30.76	16.48	9.35	13.25	14.72
	*P*-value	<0.001	<0.001	<0.001	<0.001	<0.001
** Parity: Secondiparous**	Estimate	5.80	−0.96	1.20	0.39	−3.91
	SE	0.88	0.47	0.12	0.26	1.78
	*t*-value	6.59	−2.04	10.37	1.52	−2.20
	*P*-value	<0.001	0.04	<0.001	0.13	0.03
** Parity: Primiparous**	Estimate	−0.33	−4.22	2.00	1.57	8.96
	SE	1.35	0.74	0.19	0.43	2.86
	*t*-value	−0.25	−5.73	10.31	3.67	3.14
	*P*-value	<0.001	<0.001	<0.001	<0.001	0.002
** Summer**	Estimate	5.22	1.16	−0.01	−0.19	−5.75
	SE	0.32	0.17	0.04	0.09	0.62
	*t*-value	16.40	6.81	−0.29	−2.00	−9.24
	*P*-value	<0.001	<0.001	0.77	0.05	<0.001
** Autumn**	Estimate	−1.17	−0.12	−0.02	0.07	0.79
	SE	0.22	0.11	0.03	0.06	0.41
	*t*-value	−5.41	−1.03	−0.66	1.08	1.91
	*P*-value	<0.001	0.31	0.51	0.28	0.06
** Winter**	Estimate	5.15	0.26	0.08	−0.18	−5.44
	SE	0.59	0.31	0.07	0.17	1.13
	*t*-value	8.74	0.83	1.11	−1.03	−4.81
	*P*-value	<0.001	0.41	0.27	0.30	<0.001
**Smooth terms**						
** s(Δ) multiparous**	edf	4.79	2.78	2.35	7.14	2.88
	*F*	60.46	5.29	5.56	7.41	10.42
	*P*-value	<0.001	0.001	0.002	<0.001	<0.001
** s(Δ) secondiparous**	edf	4.78	2.43	2.29	8.01	2.77
	*F*	34.07	7.08	5.00	9.66	12.10
	*P*-value	<0.001	<0.001	0.002	<0.001	<0.001
** s(Δ) primiparous**	edf	4.69	2.23	1.28	4.08	1.01
	*F*	13.44	4.34	2.38	2.72	0.68
	*P*-value	<0.001	0.01	0.07	0.02	0.41
** s (days in milk) multiparous**	edf	6.92	3.84	2.48	1.32	3.95
	*F*	154.31	23.13	22.16	99.61	26.81
	*P*-value	<0.001	<0.001	<0.001	<0.001	<0.001
** s (days in milk) secondiparous**	edf	6.68	1.00	2.77	3.59	3.95
	*F*	60.81	25.40	82.20	32.69	47.79
	*P*-value	<0.001	<0.001	<0.001	<0.001	<0.001
** s (days in milk) primiparous**	edf	6.45	1.00	2.70	3.36	3.08
	*F*	14.44	1.08	5.07	13.30	10.38
	*P*-value	<0.001	0.30	0.003	<0.001	<0.001
**Model**						
	Adjusted *R*^2^	0.80	0.78	0.85	0.83	0.79
	Deviance explained	80.8%	78.7%	85.2%	83.3%	79.0%

**Table 4:** Results of GAMM models on behavioral parameters for parametric coefficients (estimates, standard errors (SE), *t*-values, and *P*-values for parity and season fixed effects), and for smooth terms (effective degrees of freedom (edf), F-statistics, and *P*-values). For each model, the adjusted *R*^2^ and the deviance explained were reported. In the section on parametric coefficients, the estimates referred to the difference from a reference: multiparous for parity effect and spring for season effect.

		Lying time as min	Lying time as %	Daily steps	Bout number	Lying time per bout
**Parametric coefficients**						
** Intercept**	Estimate	673.87	46.80	2719.80	13.48	55.88
	SE	23.32	1.62	136.14	0.64	2.83
	*t*-value	28.90	28.90	19.98	20.94	19.75
	*P*-value	<0.001	<0.001	<0.001	<0.001	<0.001
** Parity: Secondiparous**	Estimate	16.21	1.13	267.94	−1.78	5.22
	SE	25.00	1.74	152.00	0.67	2.88
	*t*-value	0.65	0.65	1.76	−2.66	1.81
	*P*-value	0.52	0.52	0.08	0.01	0.07
** Parity: Primiparous**	Estimate	−52.14	−3.62	343.09	2.22	−17.16
	SE	36.17	2.51	214.74	1.00	4.37
	*t*-value	−1.44	−1.44	1.60	2.22	−3.93
	*P*-value	0.15	0.15	0.11	0.03	<0.001
** Summer**	Estimate	30.76	2.14	−132.53	−1.75	7.29
	SE	11.46	0.80	76.82	0.31	1.28
	*t*-value	2.68	2.68	−1.73	−5.68	5.68
	*P*-value	0.01	0.01	0.08	<0.001	<0.001
** Autumn**	Estimate	52.44	3.64	−214.46	0.20	5.60
	Standard error	7.73	0.54	52.07	0.21	0.87
	*t*-value	6.78	6.78	−4.12	0.94	6.45
	*P*-value	<0.001	<0.001	<0.001	0.35	<0.001
** Winter**	Estimate	42.90	2.98	−268.98	−0.29	4.64
	SE	19.82	1.38	129.63	0.53	2.24
	*t*-value	2.17	2.17	−2.08	−0.54	2.07
	*P*-value	0.03	0.03	0.04	0.59	0.04
**Smooth terms**						
** s(Δ) multiparous**	edf	3.91	3.91	3.80	4.64	2.79
	*F*	33.16	33.16	10.36	7.97	8.44
	*P*-value	<0.001	<0.001	<0.001	<0.001	<0.001
** s(Δ) secondiparous**	edf	3.34	3.34	3.87	2.41	1.01
	*F*	7.12	7.12	26.51	4.82	12.79
	*P*-value	<0.001	<0.001	<0.001	0.002	<0.001
** s(Δ) primiparous**	edf	3.93	3.93	3.33	2.94	2.91
	*F*	43.80	43.80	7.55	3.54	12.21
	*P*-value	<0.001	<0.001	<0.001	0.01	<0.001
** s (days in milk) multiparous**	edf	2.27	2.27	1.00	1.00	1.00
	*F*	2.58	2.58	18.53	8.68	5.04
	*P*-value	0.04	0.04	<0.001	0.003	0.02
** s (days in milk) secondiparous**	edf	2.38	2.38	2.31	2.90	2.94
	*F*	13.38	13.38	12.15	7.08	31.88
	*P*-value	<0.001	<0.001	<0.001	<0.001	<0.001
** s (days in milk) primiparous**	edf	2.26	2.26	2.39	2.91	2.70
	*F*	17.47	17.47	7.42	14.48	14.21
	*P*-value	<0.001	<0.001	0.002	<0.001	<0.001
**Model**						
	Adjusted *R*^2^	0.55	0.55	0.44	0.54	0.62
	Deviance explained	56.0%	56.0%	48.6%	55.2%	62.9%

### Changes in milk yield and milking traits

The first two figures illustrate the trends of the milking traits around the onset of lameness. In Figure 1, it is possible to observe a decrease in daily milk production across all three classes of parity (primiparous, secondiparous, and multiparous cows), starting around 16–12 d before lameness detection and treatment and ending 2–6 d after. The decrease was substantial, with milk losses of 8% (from 28.7 to 26.3 kg), 12% (from 29.0 to 25.5 kg), and 16% (from 30.3 to 25.5 kg), for primiparous, secondiparous, and multiparous cows, respectively. Following lameness resolution, from Δ  + 7/+12 d to Δ  + 19/+22 d, an increase in individual milk yield occurred, reaching 28.0 kg for primiparous cows, 27.6 kg for secondiparous, and 26.5 kg for multiparous cows, even if values remained lower than the one before the lameness event. Lameness significantly reduced milking duration by 3% for primiparous and multiparous cows and by 6% for secondiparous ones, in the slot interval from 19 to 12 d before and 1–4 d after hoof-trimmer intervention. After this period, only multiparous cows showed signals of recovery, with an 8% increase in milking duration. Average milk flow did not show any changes throughout the entire period in primiparous cows. In secondiparous cows, changes were observed only after hoof-trimming (between Δ  + 8 and Δ  + 30 d), with an increase from 1.44 to 1.59 kg/min (+10%). For multiparous cows, the decline phase occurred between Δ −26 d and Δ  +6 d, with a total decrease of 7%.

Figure 2 shows the effect of lameness on milk production emitted in the first 2 min of milking, expressed both as kg and as a percentage. For primiparous cows, milk in the first 2 min, expressed as kg, decreased by 2% between Δ −8 d and Δ −5 d. On the other hand, when expressed as a percentage, no changes were evident for primiparous cows throughout the period. For secondiparous and multiparous cows, the trend in milk production in the first 2 min (as kg) was similar, with a first drop in the 10 d before lameness (approximately −0.5 kg) followed by an increase in the 10 d after treatment (+0.6 and +0.3 kg, for secondiparous and multiparous cows, respectively). An increasing trend in the percentage of milk produced in the first 2 min was observed in secondiparous and multiparous cows, rising from 28% to 32% and from 29% to 32%, respectively, during the slot period overlapping with the lameness episode. Moreover, for multiparous cows, a drop from 31% to 30% was observed after Δ  +26 d.

The results of the GAMM models for the smooth term of Δ for milk flow in the intervals 0–15 s, 15–30 s, 30–60 s, and 60–120 s are reported in Figure S3. Between all the intervals, milk flow between 60 and 120 s showed the greatest variation before and after the lameness episode.

### Changes in lying behavior and activity patterns

Lying time, expressed as minutes and as a percentage, is reported in Figure 3. The plot of the smooth term of Δ appeared similar across the three parity classes, with an increase before lameness, followed by a decrease in the days after the hoof-trimming. Moreover, the changing points for lying time as minutes and as a percentage were the same within each parity class. For primiparous cows, the increase in lying time was observed between −17 d and +3 d of Δ, followed by a decrease between +8 d and +20 d of Δ. Lying time increased by 157 min and 11% approaching lameness, and decreased by 57 min and 4% after hoof trimming. In secondiparous cows, lying time increased by 36 min between Δ −19 d and Δ −5 d, and decreased by 16 min and 2% between +8 and +16 d of Δ. Finally, for multiparous cows, the lying time increase was 112 min and 8% from Δ −19 d to Δ  + 1 d. The consequent decrease was 60 min and 6%.

The results of the GAMM models for the smooth term of Δ for step number, bout number, and lying time per bout are reported in Figure 4. For primiparous cows, a decrease of 310 steps (−11%) was observed between Δ −14 d and Δ  +3 d. The number of bouts did not change with the approach of lameness, but the lying time per bout rose by 15% (8 min, from 52 to 60 min) during Δ −12 and Δ  +11 d and then decreased by 4 min between Δ  +19 and +30 d. For secondiparous cows, in the time interval Δ −17 and Δ  + 1 d, the number of steps increased by 29% (693 steps) and subsequently decreased by 300 steps (from Δ  +8 d to Δ  +20 d). The number of bouts dropped by 5% (from 13.6 to 12.9) after the hoof-trimmer intervention, and the lying time per bout increased by more than 7 min throughout the entire period. For multiparous cows, in the period between Δ -18 and Δ −3 d, both the number of steps and the number of bouts increased by 11% (280 steps) and 12% (from 12.8 to 14.2 bouts), respectively. Subsequently, all indicators decreased between Δ  + 1 d and Δ  + 18 d: −12% for steps and −6% for bout numbers. Lying time per bout showed a similar trend, although in a different time window (during Δ −14/0 d and during Δ 11/30 d), with an increase of 4.5% followed by a drop of 11%.

## Discussion

The farm involved in this study was considerably larger compared to the mean size of 208 adult cows per herd of the Lombardy Region and of 104 adult cows per herd of the entire Italy (AIA 2024). The low milk production detected for the sampled animals (compared to the average values of Northern Italy, Bellingeri et al. 2019) was due to the specific selection of lame cows. As highlighted by Green et al. (2002), lame cows generally produce less milk than healthy ones. Regarding the other milking traits, milking duration is comparable with the range of values reported by Mangalis et al. (2019). Average milk flow is affected by several factors, such as parity, DIM, milk production, and pre-milking procedures (Sandrucci et al. 2007); for this reason, comparisons with other studies are complex. Nevertheless, the registered values of this study align with findings of (Tancin et al. 2006) and Sandrucci et al. (2007). For behavioral indicators, the data highlighted that cows spent around 12 h lying, in line with their behavioral needs (Munksgaard et al. 2005) and with values reported by Maselyne et al. (2017). The values of lying bout number were also comparable to the findings of Gomez and Cook (2010), while the number of steps was within the ranges reported in the review by Shepley et al. (2020).

### Changes in milk yield and milking traits

As shown in the different figures presented in this study, most traits noticeably changed in trends during the period preceding lameness detection. Daily milk production began to decrease from 16 to 12 d before lameness detection. This finding aligns with previous reports showing that lame cows usually have lower milk production compared to healthy ones (Green et al. 2002). Kocak and Ekiz (2006) and Bicalho et al. (2008) reported differences of 3 kg/d between lame and healthy cows. The decrease could be due to the altered time budget of lame cows, which spent more time lying (Weigele et al. 2018), as also observed in this study. Consequently, they spent less time feeding (Miguel-Pacheco et al. 2014) and ruminating (Beer et al. 2017), causing the drop in milk yield. The extent of the effect of lameness was not the same for all the parity classes, with multiparous cows showing the greater reduction. The same finding was also reported by Warnick et al. (2001), who observed greater milk losses in cows in their second or later lactation compared to primiparous ones. The reason could be that multiparous cows may be more susceptible to lameness (Solano et al. 2015), and usually they are relapsing (Bran et al. 2018). Moreover, since multiparous cows generally produce more milk than primiparous ones, the effect of lameness is likely more marked.

The recovery from lameness, if properly treated and of moderate severity, usually occurs within a few weeks (Thomsen et al. 2019). However, as suggested by Sulayeman and Fromsa (2012), milk production may not return to pre-lameness values, as also observed in this study. In fact, milk losses often extend beyond the weeks immediately surrounding the lameness episode and may persist throughout the remainder of lactation (Warnick et al. 2001; Bicalho et al. 2008).

Milking duration also decreased as lameness approached, as suggested by Juozaitienė et al. (2021). Lame cows tend to move more during milking (Schönberger et al. 2023), potentially increasing cluster oscillations and detachment. In a rotary milking parlor where only limited supervision is possible, especially with few operators, such disturbances may be unnoticed, possibly resulting in incomplete milkings. In addition, part of the variation of milking duration (10–23%) can be explained by milk production itself (Berry et al. 2013), which helps to justify the observed decrease. On the other hand, the magnitude of the decrease in milking duration was smaller than the reduction in milk production (3–6% vs 8–16%). The reason is that milking duration is influenced not only by milk production but also by milk flow (Berry et al. 2013).

In fact, in multiparous cows, it is possible to observe a decreasing trend of milk flow with the approach, highlighting a slower milk ejection. Some studies have reported that with lower milk production, milk flow becomes slower, or emission is disturbed (Sandrucci et al. 2007). Another reason could be that the alveolar fraction of milk is emitted more slowly. Cows subjected to pain produce more adrenaline and cortisol (Zoltick et al. 2024), as in stressful situations, such as a stressful milking environment. Bruckmaier (2005) noticed that when cows are milked in unfamiliar environments, the quantity of released oxytocin is low because other hormones and physiological responses, such as adrenal cortex activity, reduce the oxytocin release. Oxytocin is the hormone that regulates alveolar milk ejection by contracting myoepithelial cells (Svennersten-Sjaunja and Olsson 2005). Therefore, pain and stress could cause disturbed milk ejection.

In fact, Juozaitienė et al. (2021) reported a higher concentration of cortisol in lame cows than in healthy ones. For primiparous and secondiparous cows, it was not possible to observe a clear trend in milk flow before lameness detection. For primiparous cows, one explanation could be that, in the first weeks after calving, the ejection of milk is sometimes disturbed (Bruckmaier 2005), so the effect of lameness may have been less evident compared to multiparous cows, which usually have, in normal conditions, regular milk ejection.

Moreover, milk production in the first 2 min, expressed as kg, decreased before lameness detection. Juozaitienė et al. (2021) also suggested that lame cows reach the plateau phase of milk ejection more slowly than healthy ones, and Wieland et al. (2022) highlighted that lameness is associated with delayed milk ejection. Therefore, alveolar milk may be emitted more slowly and with a disturbed pattern. In addition, the milk ejected in the first minutes of milking is mathematically composed of cisternal milk, which is emitted immediately after cluster attachment (Lollivier et al. 2002), and alveolar milk, which becomes available between 40 s and 2 min after teat stimulation (Bruckmaier and Wellnitz 2008). The quantity of cisternal milk is generally stable, since it is positively correlated with the interval between milkings, which was constant in this study, and with the cistern size (Ayadi et al. 2003). Supporting the previous interpretation, the missing fraction was probably alveolar milk, which may have been emitted more slowly. Regarding the percentage of milk emitted in the first 2 min of milking, an increasing trend was observed as lameness gets closer. From a mathematical point of view, this can be explained by the fact that although the absolute amount of milk in the first 2 min decreased, the total milk production decreased even more. Based on the previous considerations about alveolar milk ejection, the emission was not only disturbed and delayed but also quantitatively lower, as described by Bruckmaier (2005) in unfamiliar environments. Moreover, it is important to consider that in cases of reduced dry matter intake due to less feeding time, as occurs in lame cows (Miguel-Pacheco et al. 2014; Norring et al. 2014), probably the most affected fraction is likely alveolar milk, since the cisternal milk fraction remains constant.

The differences in the magnitude of decrease and subsequent increase of milk production in the first 2 min, expressed both as kg and as percentage, across parity classes could be attributed to differences in udder physiology. In fact, primiparous cows store less milk in the cistern than multiparous ones (Pfeilsticker et al. 1996). Moreover, they often exhibit disturbed milk ejection during the first week after calving (Bruckmaier 2005). Finally, the amount of milk stored in the alveoli depends on both the number and activity of secretory cells in the udder (Boutinaud et al. 2004).

Moreover, the theories about the delay of alveolar milk ejection or the disturbance of milk ejection with the approach of lameness remain hypotheses, as technologies capable of continuously recording the milk flow or generating the emission curves, such as those used by Sandrucci et al. (2007), were not used in this study. In addition, no hormonal data such as cortisol, adrenaline and oxytocin were collected during milking.

### Changes in lying behavior and activity patterns

For behavioral indicators, the results for lying time were consistent with the reviews of Olechnowicz and Jaskowski (2011) and Sadiq et al. (2017), reporting longer lying time in lame cows compared to the healthy ones. In our study, the increase in lying time with the approach of lameness was approximately 2 h, corresponding to 10% of the total daily time budget in both primiparous and multiparous cows. This is likely due to reduced activity of lame cows (Frondelius et al. 2022) due to the pain when moving. Miguel-Pacheco et al. (2014) reported a decrease in the number of milkings per day in lame cows milked with automatic and voluntary milking systems. In the present study, lying time began to increase around 19–16 d before lameness detection, followed by a recovery phase. Although lying time decreased after treatment, the final time spent lying remained higher than before the onset of lameness. One possible explanation is that, even when lameness is treated, the recovery time required to restore normal activity levels may exceed 30 d.

The observed decrease in step number in primiparous cows before lameness detection aligns with their reduced mobility due to discomfort. Interestingly, secondiparous and multiparous cows showed increased steps around lameness onset, possibly reflecting frequent weight shifting to relieve pain from the affected limb. Such compensatory movements are well-documented (Pastell et al. 2008; Chapinal and Tucker 2012) and likely reflect increased restlessness, especially during forced standing periods, such as milking (Schönberger et al. 2023). After recovery, step numbers returned to normal levels.

Finally, the number of bouts and the lying time per bout were considered. As supported by the study of Ito et al. (2010), severely lame cows have a higher number of bouts than healthy ones. This is consistent with the findings of the present study for multiparous cows, which exhibited an increasing trend in bout number as lameness approached. Increased bout frequency in multiparous cows may reflect restlessness due to discomfort or pain. On the other hand, lying time per bout increased across all parity classes. In particular, the increase in lying time was greater in magnitude than the increase in the number of bouts for multiparous cows. For the other parity classes, the number of bouts did not change with lameness approach.

The differences in trends of behavioral indicators between primiparous and multiparous cows were probably due to varying levels of pain and severity. As previously discussed, multiparous cows are more susceptible to recurrent lameness (Solano et al. 2015; Bran et al. 2018).

### Limitations of the study

While the present study attempted to examine the relationship between milking traits and the behavior of dairy cows concerning lameness events, it is subject to certain limitations. Notably, the data were collected from a single farm, which may restrict the generalizability of the findings. Furthermore, different types of lameness may exert varying effects, warranting further investigation in future research. Additionally, it should be noted that some traits, particularly flow variables and bout number, were modeled under the Gaussian assumption to simplify result interpretation. Although model performance metrics did not significantly differ between the Gaussian distribution and alternatives such as Tweedie or zero-inflated Poisson, results might have varied with a dataset comprising a significantly larger number of cows. Therefore, readers are encouraged to consider these aspects. Collectively, these factors may have constrained the internal and external validity of the results.

## Conclusions

This study highlighted that both milking traits and behavioral indicators began to change several days before lameness became clinically evident to the farmer. In particular, early signs of lameness were detectable at least 20–10 d before lameness detection, suggesting the potential for anticipatory monitoring. Differences in the dynamics of these traits across parity classes indicate that the biological response to lameness may vary with physiological status. This aspect needs further investigation as it may improve the individualization and accuracy of early warning systems. Although some of the observed changes were of limited amplitude, their consistency and timing suggest that they could serve as useful indicators when combined in a multivariate predictive model. Importantly, all data used in this study were collected through standard on-farm systems (milking parlor sensors and pedometers), without requiring the installation of additional devices. These findings provide a basis for the development of early detection tools based entirely on routinely available data. Future work should focus on the training and validation of machine learning algorithms capable of integrating milking and behavioral information to flag cows at risk of developing lameness before clinical signs appear, thus enabling timely intervention and improving animal welfare and productivity.

## Supplementary Material

skag289_Supplementary_Data

## Abbreviations

Δ: time distance in days between the day of observation (for milking traits and behavioral indicators) and the day of lameness detection
GAMM: Generalized Additive Mixed Model
