# Supplementary material for: Early signs of lameness in dairy cows revealed by milking and behavioral data using generalized additive mixed models
Source: J Anim Sci. 2026 Sep 8;104:skag289. doi: 10.1093/jas/skag289 (PMC13628271; doi:10.1093/jas/skag289)
Supplement: Supplementary file 1 [file skag289_supplementary_data.docx]

**Supplementary materials**

**FIGURE**

**Figure SM1**

Residual diagnostic plots using gam.check for each milking and behavioral parameter, assuming a Gaussian distribution of data.

Individual daily milk production

Milking duration

Average milk flow

Milk in first 2 minutes, kg

Milk in first 2 minutes, %

Milk flow 0-15 s

Milk flow 15-30 s

Milk flow 30-60 s

Milk flow 60-120 s

Lying time, min

Lying time, %

Daily steps

Bout number

Lying time per bout

**Figure SM2**

Residual diagnostic plots using gam. check for some milking and behavioral parameters, assuming a Tweedie distribution of data for milk flow 0-15s, and a zero-inflated Poisson distribution for bout number and daily steps.

Milk flow 0-15 s

Daily steps

Bout number

**Figure SM3**

Effect of Δ (time distance between the observation day and the day of lameness detection) on milk flow in the milking time 0-15 s, 15-30s, 30-60s and 60-120 s as fitted in a generalized mixed model for the three parity classes (primiparous, secondiparous and multiparous). The k represented the number of initial functions used for the construction of the function. P identified the P-value of the smooth term. The blue shadows showed the 95% confidence interval for each point of the smooth. The red and green lines identified the changing point of the smooth, where the derivatives of the function were statistically different from 0 in the 95% confidence interval.

**TABLES**

**Table SM1**

Comparison of deviance explained by models using individual daily milk production as addiction smooth term (with) and by the models proposed in the Materials and methods (without).

|  | Deviance explained without milk production as a smooth term | Deviance explained with milk production as a smooth term | Differences deviance explained |
| --- | --- | --- | --- |
| Milking duration | 78.7% | 79.6% | 0.9% |
| Average milk flow | 85.2% | 87.6% | 2.4% |
| Milk in first 2 minutes as kg | 83.3% | 86.1% | 2.8% |
| Milk in first 2 minutes as % | 79.0% | 79.6% | 0.6% |
| Milking flow 0-15s | 63.1% | 64.6% | 1.5% |
| Milking flow 15-30s | 81.2% | 82.8% | 1.6% |
| Milking flow 30-60s | 76.9% | 78.2% | 1.3% |
| Milking flow 60-120s | 79.8% | 82.7% | 2.9% |

**Table SM2**

The deviance explained and the AIC of models run for milk flow 0-15s, daily steps, and bouts number using the Gaussian distribution and other distributions (Tweedie for milk flow 0-15s and zero-inflated Poisson for behavioural parameters).

|  | AIC using Gaussian distribution | AIC using Tweedie (for milk flow) or zero-inflated Poisson (for behavioural) distributions | Deviance explained using Gaussian distribution. | Deviance explained using Tweedie (for milk flow) or zero-inflated Poisson (for behavioural) distributions. |
| --- | --- | --- | --- | --- |
| Milking flow 0-15 | 742 | 2 | 63.10% | 65.30% |
| Bouts number | 17330 | 17418 | 55.20% | 55.20% |
| Daily step | 55648 | 570126 | 42.90% | 48.60% |

**Table SM3**

Results of GAMM models on milking parameters for parametric coefficients (estimates, standard errors (SE), t-values, and p-values for parity and season fixed effects), and for smooth terms (effective degrees of freedom (edf), F-statistics, and p-values). For each model the adjusted R^2^ and the deviance explained were reported. In the section of parametric coefficients, the estimates referred to the difference from a reference: multiparous for parity effect and spring for season effect.

|  |  | Milking flow 0-15s | Milking flow 15-30s | Milking flow 30-60s | Milking flow 60-120s |
| --- | --- | --- | --- | --- | --- |
| Parametric coefficients | |  |  |  |  |
| Intercept | Estimate | 0.24 | 1.74 | 1.80 | 2.83 |
|  | SE | 0.06 | 0.19 | 0.16 | 0.20 |
|  | t-value | 4.25 | 8.93 | 11.16 | 13.95 |
|  | P-value | <0.001 | <0.001 | <0.001 | <0.001 |
| Parity: Secondiparous | Estimate | 0.04 | 0.89 | -0.03 | -0.05 |
|  | SE | 0.06 | 0.16 | 0.16 | 0.18 |
|  | t-value | 0.59 | 5.50 | -0.18 | -0.27 |
|  | P-value | 0.56 | <0.001 | 0.86 | 0.79 |
| Parity: Primiparous | Estimate | -0.08 | 1.10 | 0.23 | 0.75 |
|  | SE | 0.09 | 0.27 | 0.24 | 0.30 |
|  | t-value | -0.95 | 4.04 | 0.96 | 2.52 |
|  | P-value | 0.34 | <0.001 | 0.34 | 0.01 |
| Summer | Estimate | 0.04 | -0.26 | -0.22 | 0.01 |
|  | SE | 0.03 | 0.06 | 0.06 | 0.07 |
|  | t-value | 1.43 | -4.22 | -3.48 | 0.09 |
|  | P-value | 0.15 | <0.001 | <0.001 | 0.93 |
| Autumn | Estimate | 0.08 | 0.05 | 0.08 | -0.02 |
|  | SE | 0.02 | 0.04 | 0.04 | 0.05 |
|  | t-value | 4.41 | 1.21 | 1.90 | -0.51 |
|  | P-value | <0.001 | 0.23 | 0.06 | 0.61 |
| Winter | Estimate | 0.06 | -0.18 | -0.34 | 0.09 |
|  | SE | 0.05 | 0.11 | 0.11 | 0.13 |
|  | t-value | 1.34 | -1.57 | -3.08 | 0.67 |
|  | P-value | 0.18 | 0.12 | 0.00 | 0.50 |
| Smooth therms | |  |  |  |  |
| s(Δ) multiparous | edf | 3.44 | 2.46 | 2.18 | 7.24 |
|  | F | 6.59 | 1.65 | 3.41 | 7.31 |
|  | P-value | <0.001 | 0.17 | 0.03 | <0.001 |
| s(Δ) secondiparous | edf | 1.00 | 6.96 | 2.89 | 7.95 |
|  | F | 0.18 | 6.32 | 17.84 | 7.85 |
|  | P-value | 0.67 | <0.001 | <0.001 | <0.001 |
| s(Δ) primiparous | edf | 1.00 | 4.21 | 2.50 | 4.96 |
|  | F | 0.93 | 1.77 | 1.50 | 3.68 |
|  | P-value | 0.34 | 0.12 | 0.22 | 0.00 |
| s(days in milk) multiparous | edf | 2.02 | 1.00 | 2.57 | 1.00 |
|  | F | 1.17 | 183.52 | 114.22 | 21.92 |
|  | P-value | 0.20 | <0.001 | <0.001 | <0.001 |
| s(days in milk) secondiparous | edf | 2.43 | 1.81 | 2.57 | 3.94 |
|  | F | 11.87 | 31.42 | 17.39 | 19.41 |
|  | P-value | <0.001 | <0.001 | <0.001 | <0.001 |
| s(days in milk) primiparous | edf | 1.00 | 2.61 | 2.71 | 6.34 |
|  | F | 1.14 | 11.53 | 13.81 | 6.52 |
|  | P-value | 0.29 | <0.001 | <0.001 | <0.001 |
| Model |  |  |  |  |  |
|  | Adjusted R2 | 0.62 | 0.81 | 0.76 | 0.79 |
|  | Deviance explained | 63.1% | 81.2% | 76.9% | 79.8% |

**Table SM4**

Mean derivative, lower CI derivative and upper CI derivative of changing points of each milking and behavioural parameter.

|  | Primiparous | | | | Secondiparous | | | | Multiparous | | | |
| --- | --- | --- | --- | --- | --- | --- | --- | --- | --- | --- | --- | --- |
| Parameters | Δ | Derivative | Lower CI | Upper CI | Δ | Derivative | Lower CI | Upper CI | Δ | Derivative | Lower CI | Upper CI |
| Milk Production | -12 | -0.118 | -0.213 | -0.023 | -11 | -0.089 | -0.174 | -0.004 | -16 | -0.081 | -0.150 | -0.012 |
|  | 2 | -0.066 | -0.154 | 0.023 | 6 | -0.061 | -0.128 | 0.005 | 6 | -0.052 | -0.112 | 0.009 |
|  | 7 | 0.089 | 0.014 | 0.164 | 10 | 0.104 | 0.021 | 0.186 | 12 | 0.087 | 0.005 | 0.168 |
|  | 19 | 0.064 | -0.012 | 0.139 | 22 | 0.089 | -0.008 | 0.187 | 21 | 0.060 | -0.012 | 0.132 |
| Average milk flow | NA |  |  |  | 8 | 0.004 | 0.0002 | 0.008 | -26 | -0.006 | -0.013 | -0.0001 |
|  |  |  |  |  | 30 | 0.008 | 0.001 | 0.015 | -5 | -0.003 | -0.007 | 0.0000 |
| Milking duration | -12 | -0.017 | -0.0325 | -0.00148 | -19 | -0.027 | -0.052 | -0.001 | -11 | -0.016 | -0.031 | -0.001 |
|  | 3 | -0.017 | -0.0344 | 0.000308 | 2 | -0.018 | -0.037 | 0.0003 | 1 | -0.020 | -0.040 | 0.0004 |
|  |  |  |  |  |  |  |  |  | 13 | 0.017 | 0.001 | 0.032 |
|  |  |  |  |  |  |  |  |  | 30 | 0.049 | 0.016 | 0.082 |
| Milk in first 2 minutes as kg | -8 | -0.028 | -0.055 | -0.0004 | -8 | -0.072 | -0.127 | -0.017 | -10 | -0.053 | -0.093 | -0.012 |
|  | -5 | -0.027 | -0.054 | 0.001 | -2 | -0.033 | -0.085 | 0.019 | -3 | -0.037 | -0.078 | 0.004 |
|  |  |  |  |  | 1 | 0.059 | 0.003 | 0.114 | 2 | 0.051 | 0.009 | 0.092 |
|  |  |  |  |  | 8 | 0.052 | -0.005 | 0.108 | 7 | 0.036 | -0.009 | 0.082 |
| Milk in first 2 minutes as % | NA |  |  |  | -12 | 0.079 | 0.015 | 0.143 | -11 | 0.068 | 0.014 | 0.122 |
|  |  |  |  |  | 16 | 0.072 | -0.014 | 0.158 | 11 | 0.051 | -0.001 | 0.103 |
|  |  |  |  |  |  |  |  |  | 26 | -0.120 | -0.240 | -0.001 |
|  |  |  |  |  |  |  |  |  | 30 | -0.125 | -0.247 | -0.003 |
| Milk flow 0-15 s | NA |  |  |  | NA |  |  |  | -24 | -0.008 | -0.016 | -0.0002 |
|  |  |  |  |  |  |  |  |  | -16 | -0.005 | -0.011 | 0.0001 |
| Milk flow 15-30 s | NA |  |  |  | -7 | -0.032 | -0.063 | -0.001 | NA |  |  |  |
|  |  |  |  |  | -1 | -0.025 | -0.055 | 0.005 |  |  |  |  |
|  |  |  |  |  | 4 | 0.033 | 0.004 | 0.061 |  |  |  |  |
|  |  |  |  |  | 11 | 0.021 | -0.009 | 0.050 |  |  |  |  |
| Milk flow 30-60 s | NA |  |  |  | -15 | -0.009 | -0.017 | -0.001 | -18 | -0.007 | -0.015 | -0.00001 |
|  |  |  |  |  | 4 | -0.008 | -0.016 | 0.0003 | -9 | -0.005 | -0.009 | 0.0001 |
|  |  |  |  |  | 11 | 0.007 | 0.0003 | 0.013 |  |  |  |  |
|  |  |  |  |  | 30 | 0.031 | 0.016 | 0.045 |  |  |  |  |
| Milk flow 60-120 s | -9 | -0.03 | -0.05 | -0.001 | -8 | -0.049 | -0.088 | -0.009 | -10 | -0.035 | -0.064 | -0.006 |
|  | -4 | -0.02 | -0.05 | 0.001 | -3 | -0.031 | -0.067 | 0.004 | -2 | -0.018 | -0.049 | 0.013 |
|  |  |  |  |  | 1 | 0.054 | 0.015 | 0.093 | 2 | 0.036 | 0.006 | 0.067 |
|  |  |  |  |  | 7 | 0.040 | -0.001 | 0.081 | 7 | 0.031 | -0.002 | 0.064 |
| Lying time as min | -17 | 2.26 | 0.37 | 4.14 | -19 | 2.38 | 0.28 | 4.48 | -19 | 2.34 | 0.19 | 4.49 |
|  | 3 | 2.02 | -0.17 | 4.22 | -5 | 1.69 | -0.20 | 3.57 | 1 | 0.84 | -0.72 | 2.40 |
|  | 8 | -3.45 | -6.19 | -0.72 | 8 | -2.03 | -4.06 | -0.01 | 4 | -2.07 | -3.92 | -0.23 |
|  | 20 | -2.87 | -5.90 | 0.17 | 16 | -1.64 | -3.33 | 0.05 | 19 | -1.32 | -3.29 | 0.64 |
| Lying time as % | -17 | 0.157 | 0.028 | 0.286 | -19 | 0.166 | 0.020 | 0.311 | -19 | 0.163 | 0.013 | 0.312 |
|  | 3 | 0.140 | -0.010 | 0.291 | -5 | 0.117 | -0.013 | 0.248 | 1 | 0.059 | -0.050 | 0.167 |
|  | 8 | -0.240 | -0.427 | -0.053 | 8 | -0.141 | -0.282 | -0.001 | 4 | -0.144 | -0.272 | -0.016 |
|  | 20 | -0.199 | -0.407 | 0.009 | 16 | -0.114 | -0.231 | 0.003 | 19 | -0.092 | -0.229 | 0.045 |
| Daily steps | -14 | -12.0 | -24.0 | -0.02 | -17 | 16.5 | 4.29 | 28.7 | -17 | 13.1 | 1.68 | 24.4 |
|  | 3 | -10.7 | -23.6 | 2.28 | 1 | 17.6 | 5.67 | 29.5 | -3 | 8.71 | -3.00 | 20.4 |
|  |  |  |  |  | 8 | -19.5 | -35.9 | -3.14 | 2 | -12.2 | -22.9 | -1.41 |
|  |  |  |  |  | 20 | -16.4 | -33.9 | 1.18 | 18 | -9.28 | -20.9 | 2.37 |
| Bout number | NA |  |  |  | 4 | -0.039 | -0.077 | -0.001 | -18 | 0.071 | 0.012 | 0.131 |
|  |  |  |  |  | 19 | -0.046 | -0.094 | 0.002 | -5 | 0.040 | -0.021 | 0.100 |
|  |  |  |  |  |  |  |  |  | 1 | -0.088 | -0.162 | -0.013 |
|  |  |  |  |  |  |  |  |  | 10 | -0.061 | -0.132 | 0.010 |
| Lying time per bout | -12 | 0.146 | 0.009 | 0.283 | -30 | 0.125 | 0.0516 | 0.199 | -14 | 0.137 | 0.007 | 0.267 |
|  | 11 | 0.109 | -0.031 | 0.250 | 30 | 0.125 | 0.0508 | 0.198 | 0 | 0.148 | -0.001 | 0.297 |
|  | 19 | -0.263 | -0.526 | 0.001 |  |  |  |  | 11 | -0.120 | -0.218 | -0.021 |
|  | 30 | -0.443 | -0.792 | -0.095 |  |  |  |  | 30 | -0.436 | -0.685 | -0.187 |
